# Multidimensional physiological impairments are associated with frailty and survival in older patients with cancer

**DOI:** 10.1515/jtim-2026-0057

**Published:** 2026-08-24

**Authors:** Xiangting Dai, Xi Zhang, Haihong Wang, Jiaxin Huang, Chenan Liu, Le Tian, Min Yang, Pingping Jia, Chunhua Song, Hanping Shi, Minghua Cong

**Affiliations:** Department of Comprehensive Oncology, National Cancer Center/National Clinical Research Center for Cancer/Cancer Hospital, Chinese Academy of Medical Sciences and Peking Union Medical College, Beijing, China; Key Laboratory of Cancer FSMP for State Market Regulation, Beijing, China; Beijing International Science and Technology Cooperation Base for Cancer Metabolism and Nutrition, Beijing, China; Department of Clinical Nutrition, Beijing Shijitan Hospital, Capital Medical University, Beijing, China; Department of Epidemiology, College of Public Health, Zhengzhou University, Zhengzhou, Henan Province, China

**Keywords:** cancer, prognosis, frailty, malnutrition, inflammation, survival

## Abstract

**Background and Objectives:**

Frailty, which is influenced by the intricate interactions between malnutrition, metabolism, and systemic inflammation, is highly prevalent in the elderly and constitutes a significant barrier to the effective treatment of older patients with cancer. Assessing specific biomarker patterns may enhance our understanding of the mechanisms underlying the intricate networks that contribute to frailty in this population. This study aimed to examine the association between frailty and malnutrition, metabolism, and systemic inflammation in older patients with cancer, and to further assess their prognostic value.

**Methods:**

This multicenter cohort study included 2219 older patients with cancer categorized as non-frail (*n* = 533), pre-frail (*n* = 1084), or frail (*n* = 602) according to the Fried frailty criteria at a single time point. Principal component analysis (PCA) was performed on 18 variables encompassing nutritional, metabolic, and inflammatory biomarkers to derive the principal components (PCs). Cox proportional hazards and logistic regression models were used to assess the associations between PCs, frailty, and overall survival (OS).

**Results:**

Three PCs were identified among 18 variables. PC1 represented a malnutrition-associated pattern. PC2 represented a metabolism-associated pattern. PC4 represented an inflammation-associated pattern. PC3 was excluded because of its redundancy with PC1. Frail patients exhibited significantly higher factor scores compared to pre-frail and robust patients in PC1 (median [IQR]: 1.03 [1.82] *vs*. -0.12 [1.65] *vs*. -0.93 [1.52]; *P* < 0.001), PC2 (0.20 [1.90] *vs*. -0.03 [1.29] *vs*. -0.17 [1.03]; *P* < 0.001), and PC4 (0.07 [1.27] *vs*. -0.04 [0.97] *vs*. -0.06 [0.75]; *P* = 0.049). Furthermore, patients with higher factor scores in PC1, PC2, or PC4 were significantly associated with frailty [adjusted OR: 2.13, 95% CI: 1.90–2.39, *P* < 0.001 for PC1; adjusted OR: 1.13, 95% CI: 1.03–1.23, *P* = 0.008 for PC2; adjusted OR: 1.12, 95% CI: 1.03–1.22, *P* = 0.011 for PC4]. Frail patients exhibited significantly worse survival outcomes compared to pre-frail individuals (adjusted HR = 1.67, 95% CI: 1.45–1.92; *P* < 0.001).

**Conclusions:**

Frailty is significantly associated with decreased survival in older patients with cancer and coexists with multidimensional physiological impairments, including nutritional deficits, metabolic disturbances, and systemic inflammation.

## Introduction

Cancer has emerged as a predominant health burden among the elderly, and the complex biological interplay between the aging process and oncogenesis significantly complicates clinical management.^[1]^ Research has indicated that a substantial proportion of newly diagnosed cancer cases and over 70% of cancer-related deaths occur in older adults,^[2,3]^ underscoring the profound impact of cancer on health outcomes in this population. This trend is expected to intensify with the continued global population aging, leading to a substantial increase in cancer incidence among older adults in the coming decades.

Frailty is a key geriatric syndrome characterized by diminished physiological reserves and increased vulnerability to stressors.^[4]^ Its prevalence increases with age, affecting approximately 42% of older adults with cancer.^[5]^ A recent longitudinal study elucidated distinct frailty trajectories in this population, revealing that 52.8% of older cancer survivors exhibited sustained low-risk profiles, whereas 25.0% experienced progressive worsening over a six-year period.^[6]^ Frailty has a profound negative impact on cancer prognosis. It is independently associated with adverse clinical outcomes, including increased risk of postoperative complications, chemotherapy-related toxicity, diminished health-related quality of life, and elevated mortality.^[7,8]^

Frailty and cancer share several age-related pathophysiological mechanisms, including chronic inflammation, metabolic dysregulation, and malnutrition.^[9,10]^ Chronic inflammation accelerates muscle catabolism and impairs metabolic function, whereas malnutrition compromises tissue repair and energy homeostasis. These processes collectively promote sarcopenia—a central component of frailty—contributing to a vicious cycle that accelerates cancer progression.^[11, 12, 13]^

Current frailty assessments predominantly rely on phenotypic definitions such as Fried's frailty phenotype.^[14]^ Although valuable in clinical settings, these tools provide limited insights into the underlying biological mechanisms of frailty. Nutritional, metabolic, and inflammatory statuses have been shown in multiple studies to be associated with adverse outcomes in geriatric populations.^[15,16]^ For instance, in a longitudinal study spanning eight years, elevated levels of baseline high-sensitivity C-reactive protein (hs-CRP) were found to predict worsening frailty in community-dwelling older adults.^[15]^ However, a single indicator cannot comprehensively capture the complex biological processes underlying frailty, which involve the cumulative dysregulation of multiple physiological systems, including chronic inflammation, cellular senescence, mitochondrial dysfunction, and impaired nutrient sensing.^[9,10]^ Moreover, including multiple correlated biomarkers directly in conventional regression models may lead to substantial multicollinearity and compromise model stability. Principal component analysis (PCA) can address these limitations and is therefore a necessary methodological approach.

In the present study, we conducted a systematic investigation within the framework of the Investigation on Nutrition Status and its Clinical Outcome of Common Cancers (INSCOC) program,^[17]^ focusing on the association between PCA-derived multidimensional biomarker patterns (including nutritional, metabolic, and inflammatory), frailty status, and their prognostic significance for overall survival (OS) in older adults with cancer.

## Patients and methods

### Study design and population

In this multicenter prospective cohort study, we evaluated 2219 elderly patients with cancer from the INSCOC project between January 2013 and December 2022.^[17]^ The inclusion criteria were as follows: (1) age ≥ 65 years, (2) availability of comprehensive clinical data. The exclusion criteria were as follows: (1) presence of an active infection or autoimmune disease and (2) absence of essential baseline data required for analysis. Details of the study design are shown in Supplementary Figure S1. Only data from the first admission were used for patients with multiple admissions for cancer treatment.

### Patient characteristics

Specialized personnel collected comprehensive baseline data for each participant within 48 h of hospital admission through face-to-face interviews, physical examinations, and laboratory tests. The baseline characteristics included frailty status according to Fried's frailty criteria, age, sex, body mass index (BMI), presence of chronic disease, smoking and drinking status, tea-drinking habits, tumor type, TNM stage, metastasis, neutrophil-to-lymphocyte ratio (NLR), functional status, global health status, symptom burden, and a summary score derived from the EORTC QLQ-C30 questionnaire.

### Frailty assessment and OS definition

Frailty status was assessed according to the conceptual framework of Fried's frailty phenotype,^[14]^ which includes five components: shrinking, weakness, exhaustion, slowness, and low physical activity. The operational definitions of each component used in this study are presented in Supplementary Table S1. Individuals meeting three or more of these five criteria were categorized as frail. Those classified as pre-frail exhibited one or two of these components, whereas those exhibiting none were considered non-frail.

OS was defined as the time interval from the date of enrollment to the date of death. Patients who were lost to follow-up, withdrawn from the study, or alive at the end of the follow-up were right-censored at their last known contact date.

### Biomarker inclusion and PCA

All objectively measured biochemical and anthropometric parameters available in the INSCOC database were analyzed. Specifically, 18 biomarkers—including weight loss, total protein (TP), albumin (ALB), BMI, mid-arm circumference (MAC), triceps skinfold thickness (TSF), calf circumference (CC), aspartate aminotransferase (AST), alanine aminotransferase (ALT), total bilirubin (Tbil), white blood cells (WBC), neutrophils, lymphocytes, total cholesterol (TC), low-density lipoprotein cholesterol (LDL-C), high-density lipoprotein cholesterol (HDL-C), and hand grip strength (HGS), were standardized prior to analysis. Weight loss was defined as the percentage of unintentional body weight reduction over the following 6 months, calculated as (weight at 6 months – weight at baseline) / weight at baseline × 100%.

PCA, a widely used unsupervised dimensionality-reduction technique, was used to capture the latent structures underlying a wide array of interrelated biomarkers. PCA transforms a high-dimensional biomarker space into a lower-dimensional latent space by identifying orthogonal principal components (PCs) that represent linear combinations of the original variables and maximize the explained variance. PCs were extracted based on eigenvalues > 1, scree plot inspection (Supplementary Figure S2), and biologically meaningful interpretations. Factor loadings ≥ |0.20| were considered significant to balance sensitivity and specificity and to avoid excluding biomarkers with potentially meaningful but modest biological contributions, which is appropriate given the large sample size (*n* = 2219). Three biologically meaningful PCs were identified among the 18 variables.

### Statistical analyses

Baseline patient characteristics were summarized according to their frailty status. Continuous variables are reported as medians (interquartile ranges [IQRs]), while categorical variables are presented as frequencies and percentages. Comparisons of baseline characteristics across groups were conducted using χ^2^ tests or Fisher's exact tests for categorical variables and the Mann–Whitney *U* tests for continuous variables. Logistic regression was used to evaluate the association between PCs and frailty status, reported as adjusted odds ratios (ORs) with 95% confidence intervals (CIs). Survival outcomes were analyzed using univariate and multivariate Cox proportional hazards models to estimate the hazard ratios (HRs) and 95% confidence intervals (CIs) for OS. Multicollinearity was assessed by calculating the variance inflation factors (VIFs) for all variables in the regression models. All VIFs were below the conventional threshold of five, indicating no concerning collinearity (Supplementary Table S2). Statistical analyses were performed using the R software (version 4.4.2; R Foundation for Statistical Computing, Vienna, Austria). All statistical tests were two-sided, and *p*-values < 0.05 were considered statistically significant.

### Missing data

The proportion of missing data for all baseline variables was < 5%. Participants with missing values were excluded from the analyses requiring those variables (complete-case analysis). According to established methodological guidelines, when missingness is below 5%, a complete-case analysis is considered appropriate and unlikely to bias the results.^[18,19]^ Therefore, no imputation methods were applied, and all analyses were performed on complete cases.

## Results

### Baseline characteristics of the study population

Of the 2219 older patients with cancer enrolled in the study, 533 (24.0%) were classified as non-frail, 1084 (48.9%) as pre-frail, and 602 (27.1%) as frail. The baseline characteristics are summarized in Supplementary Table S3. The frail patients were significantly older than the pre-frail and non-frail individuals, with median ages of 71.00, 69.00, and 68.00 years, respectively (*P* < 0.001). They were also more likely to have comorbid chronic diseases (54.7%, 48.6%, and 46.9%, respectively; *P* = 0.018) and to exhibit higher proportions of underweight status (BMI < 18.5 kg/m^2^) (23.6%, 11.3%, and 5.8%, *P* < 0.001), stage IV tumors (56.8%, 42.3%, and 32.5%, *P* < 0.001), metastatic disease (53.5%, 39.8%, and 29.6%, *P* < 0.001), and elevated NLR (NLR ≥ 3: 51.2%, 36.6%, and 27.8%, respectively; *P* < 0.001).

Frail patients demonstrated significantly poorer functional scores than pre-frail and non-frail individuals (median: 69.00, 86.67, and 97.33), global health status (median: 50.00, 66.67, and 83.33), symptom burden (median: 21.30, 9.88, and 3.70), and overall QoL (median: 47.26, 53.44, and 60.05). Compared to pre-frail and non-frail participants, frail patients had a higher prevalence of gastric, hepatobiliary, and pancreatic cancers. Males accounted for 63.8% of the overall population, and sex distribution did not differ significantly across the frailty groups (*P* = 0.640). Lifestyle factors, including smoking, alcohol consumption, and tea consumption, did not differ significantly between groups.

**Figure 1:**
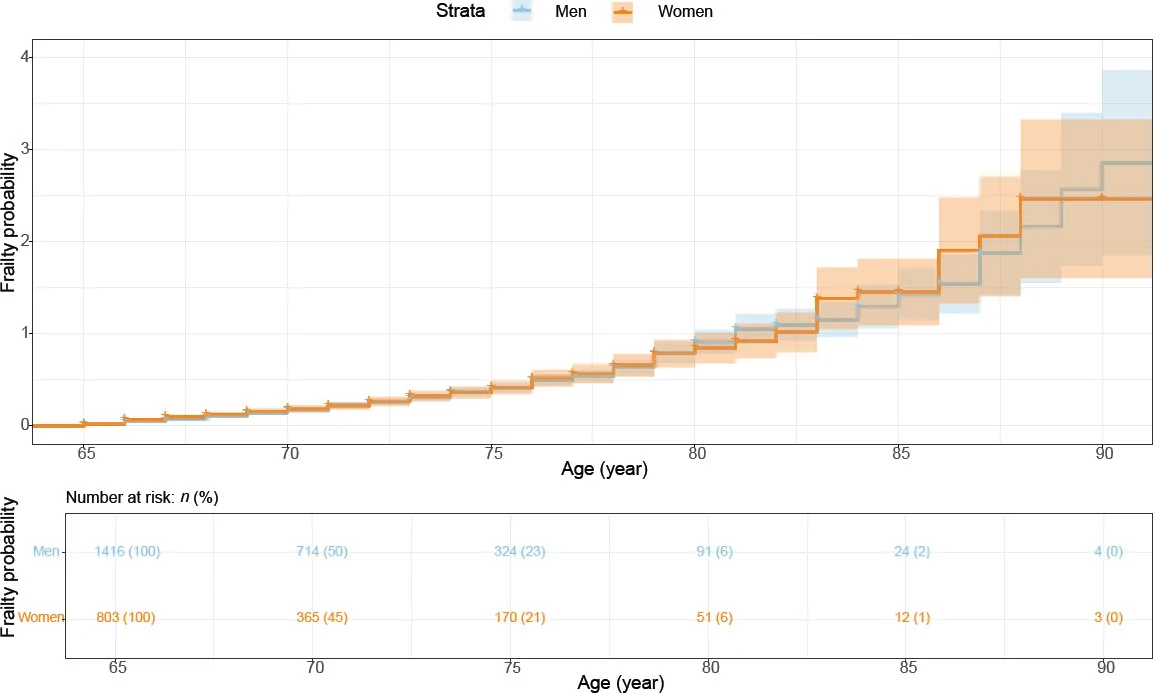
Age-dependent increase in frailty probability by sex in older patients with cancer. Frailty probability across age is presented separately for men and women using stepwise model estimates with 95% confidence intervals. The number at risk in each age group is provided beneath the figure.

Furthermore, Figure 1 illustrates the age-dependent increase in the probability of frailty, stratified by sex. The analysis demonstrated a progressive increase in the probability of frailty with advancing age in both sexes, with consistently higher probabilities in female patients across all age groups. The number of at-risk patients within each age group is shown in detail in the figure.

### Identification of biological patterns by PCA

Three biomarker patterns (PC1, PC2, and PC4) were identified through exploratory analysis (Table 1). The scree plot showed an inflection in the fourth component (Supplementary Figure S2). However, PC3 showed substantial overlap with PC1 in terms of both loading structure and biological meaning (Table 1). Because PC3 did not provide additional independent information, it was excluded from subsequent analyses to avoid redundancy. Specifically, PC1 primarily reflected a malnutrition-related pattern characterized by a strong positive loading for weight loss and negative loadings for albumin, total protein, cholesterol, LDL-C, BMI, and anthropometric measures, including MAC, TSF, and CC. Accordingly, higher PC1 factor scores were associated with a greater degree of malnutrition as reflected by the corresponding biomarker changes. PC2 reflected a metabolic pattern characterized by elevated levels of cholesterol, AST, ALT, bilirubin, and LDL-C. Higher PC2 scores are indicative of metabolic dysregulation. PC4 represents an inflammatory signature characterized by increased levels of WBC, neutrophils, and lymphocytes. Therefore, higher PC4 factor scores were associated with a heightened inflammatory state, as indicated by the corresponding biomarker levels.

**Table 1:** Principal components 1, 2 and 4 with respective factor loadings of the components among older patients with cancer

Factor	Factor loading			
	PC1	PC2	PC3	PC4
TP	-0.255	/	-0.260	/
ALB	-0.309	/	-0.246	/
TC	-0.252	0.272	-0.444	/
TG	/	/	/	/
AST	/	0.563	0.202	/
ALT	/	0.556	0.201	/
Tbil	/	0.430	/	/
HDL	/	/	-0.301	/
LDL	-0.238	0.252	-0.396	/
WBC	/	/	/	0.436
Neutrophil	/	/	/	0.648
Lymphocyte	/	/	/	0.570
BMI	-0.422	/	0.277	/
MAC	-0.417	/	0.283	/
TSF	-0.314	/	/	/
CC	-0.366	/	0.302	/
HGS	/	/	/	/
Weight loss	0.235	/	/	/

Components with factor loadings between 0.200 and -0.200 are not shown in this table; components and factor loadings in italics represent negative loading; PC1: eigenvalue = 3.281, variance = 13.95%; PC2: eigenvalue = 2.066, variance = 11.63%; PC4: eigenvalue = 1.371, variance = 7.62%. ALB: albumin; ALT: alanine aminotransferase; AST: aspartate aminotransferase; BMI: body mass index; CC: calf circumference; HDL: high-density lipoprotein; HGS: hand grip strength; LDL: low-density lipoprotein; MAC: mid-arm circumference; PC: principal component; TC: total cholesterol; Tbil: total bilirubin; TG: triglycerides; TP: total protein; TSF: triceps skinfold thickness; WBC: white blood cell.

### Associations between biomarker patterns and frailty status

**Figure 2:**
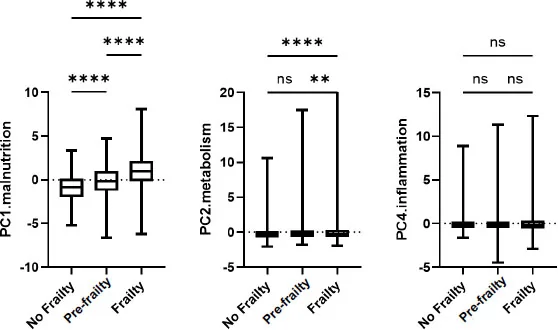
Factor scores of principal components 1, 2 and 4 by frailty status among older patients with cancer. ns, not significant; **P < 0.01; ****P < 0.0001.

Supplementary Figure S3 shows the distribution of 18 circulating biomarkers across different frailty states in older patients with cancer. Using PCA, three principal components were derived from these biomarkers. Notably, frail patients exhibited significantly higher factor scores across all PCs than pre-frail and non-frail individuals. For PC1, which represents a malnutrition-related pattern, the median (IQR) factor scores were 1.03 (1.82) in the frail group, compared to −0.12 (1.65) in the pre-frail group and −0.93 (1.52) in the non-frail group (*P* < 0.001). Similarly, PC2, reflecting a metabolic pattern, showed significantly higher factor scores in frail participants (0.20 [1.90]) compared to their pre-frail (−0.03 [1.29]) and non-frail (−0.17 [1.03]) counterparts (*P* < 0.001). PC4, indicative of an inflammation-related pattern, also showed statistically significant differences in factor scores across groups: frailty (0.07 [1.27]), pre-frailty (−0.04 [0.97]), and non-frailty (−0.06 [0.75]) (*P* = 0.049) (Figure 2).

Supplementary Figure S4 illustrates the spatial distribution of the frailty phenotypes based on the derived PCs representing the nutritional, metabolic, and inflammatory domains. The plot revealed a clear spatial gradient across frailty states. Frailty predominantly occupied regions associated with higher values along the PC1 (malnutrition) and PC2 (metabolic alteration) axes. Pre-frail individuals were generally clustered in intermediate positions, whereas non-frail patients tended to localize more frequently in regions corresponding to lower values across all three PCs. This spatial separation suggests that distinct underlying biological patterns are associated with increasing frailty severity, with malnutrition and metabolic dysregulation appearing particularly prominent in frail phenotypes.

Logistic regression analysis further demonstrated that higher scores on each PC were independently associated with frailty status. The adjusted OR was 2.13 (95% CI: 1.90–2.39; *P* < 0.001) for PC1, 1.13 (95% CI: 1.03–1.23; *P* = 0.008) for PC2, and 1.12 (95% CI: 1.03–1.22; *P* = 0.011) for PC4 (Table 2). Furthermore, we examined the potential interaction effects between the PCs (PC1 × PC2, PC1 × PC4, and PC2 × PC4). None of these interaction terms reached statistical significance, as all adjusted *P* values were greater than 0.05.

**Table 2:** Association of principal components (PCs) with frailty status in older patients with cancer

Frailty-per SD	OR (95%CI)	*P* value	Adjusted OR (95%CI)	*P* value
PC1. malnutrition	2.21 (1.99, 2.46)	<0.001	2.13 (1.90, 2.39)	<0.001
PC2. metabolism	1.18 (1.08, 1.28)	<0.001	1.13 (1.03, 1.23)	0.008
PC4. inflammation	1.10 (1.01, 1.19)	0.028	1.12 (1.03, 1.22)	0.011
PC1 × PC2 interaction	0.98 (0.89, 1.07)	0.600	0.98 (0.90, 1.07)	0.675
PC1 × PC4 interaction	0.99 (0.89, 1.10)	0.817	0.98 (0.88, 1.08)	0.642
PC2 × PC4 interaction	1.07 (0.99, 1.15)	0.092	1.05 (0.97, 1.13)	0.220

Adjusted for age, sex, chronic disease, smoking, drinking, tumor type, tumor stage, metastasis, surgery, chemotherapy, radiotherapy.

### Prognostic impact of frailty and biomarker patterns on OS

With pre-frailty as the reference, frail older adults with cancer had a higher hazard of death (adjusted HR = 1.67, 95% CI: 1.45–1.92; *P* < 0.001), whereas non-frail patients had a lower hazard (adjusted HR = 0.64, 95% CI: 0.53–0.77; *P* < 0.001). A significant linear trend in mortality risk was observed with increasing frailty severity (*P* for trend < 0.001, Table 3).

**Table 3:** Association of frailty status with overall survival (OS) in older patients with cancer

Variables	HR (95%CI)	*P* value	Adjusted HR (95%CI)	*P* value
No Frailty	0.58 (0.49, 0.70)	<0.001	0.64 (0.53, 0.77)	<0.001
Pre-frailty		reference		reference
Frailty	1.99 (1.74, 2.28)	<0.001	1.67 (1.45, 1.92)	<0.001
*P* for trend		<0.001		<0.001

Adjusted for age, sex, chronic disease, smoking, drinking, tumor type, TNM stage, metastasis, surgery, chemotherapy, and radiotherapy. TNM: tumor-node-metastasis.

The biomarker patterns identified through PCA further refined the prognostic stratification. Higher factor scores in the malnutrition-related pattern (PC1) were independently and significantly associated with increased mortality risk (adjusted HR = 1.30, 95% CI: 1.22–1.38; *P* < 0.001). Similarly, elevated scores in the metabolism-related pattern (PC2) were significantly associated with worse OS (adjusted HR = 1.11, 95% CI: 1.05–1.17; *P* < 0.001). In univariable analysis, the inflammation-related component (PC4) was modestly associated with poorer OS (HR = 1.08, 95% CI: 1.02–1.14; *P* = 0.014). After multivariable adjustment, the effect showed a trend toward significance (adjusted HR = 1.06, 95% CI: 1.00–1.12; *P* = 0.061; Supplementary Table S4). Furthermore, we assessed the potential interactions among the PCs. The interaction term between PC1 and PC2 (PC1 × PC2) was not statistically significant in either the unadjusted (*P* = 0.320) or adjusted (*P* = 0.715) models. Similarly, the interactions between PC1 and PC4 (PC1 × PC4) and PC2 and PC4 (PC2 × PC4) were not statistically significant.

### Comparison of prognostic indices

As summarized in Supplementary Table S5, multivariable Cox regression identified several significant predictors of OS, including gender (adjusted HR = 0.77, 95% CI: 0.65–0.90; *P* = 0.001), age (adjusted HR = 1.02, 95% CI: 1.01–1.03; *P* = 0.005), BMI (adjusted HR = 0.96, 95% CI: 0.94–0.97; *P* = 0.001), tumor type, TNM stage (adjusted HR = 2.02, 95%CI: 1.66–2.47; *P* = 0.001), metastasis (adjusted HR = 1.60, 95% CI: 1.36–1.87; *P* = 0.001), chemotherapy (adjusted HR = 1.25, 95% CI: 1.08–1.44; *P* = 0.003), surgery (adjusted HR = 0.72, 95% CI: 0.61–0.83; *P* = 0.001), NLR (adjusted HR = 1.31, 95% CI: 1.15–1.49; *P* = 0.001), and EORTC QLQ-C30 scores (adjusted HR = 0.97, 95% CI: 0.96–0.97; *P* = 0.001).

The C-indices for each predictor are listed in Supplementary Table S6. Among all variables, frailty (C-index = 0.641, 95%CI: 0.623–0.659), metastasis (C-index = 0.629, 95%CI: 0.613–0.645) and PC1. malnutrition (C-index = 0.620, 95%CI: 0.602–0.639) achieved higher C-indices, outperforming EORTC QLQ-C30 (C-index = 0.618, 95%CI: 0.599–0.637), BMI (C-index = 0.591, 95%CI: 0.572–0.609), NLR (C-index = 0.577, 95%CI: 0.561–0.594), and other clinical variables.

### Subgroup analyses by cancer type

Subgroup analyses were conducted for lung, gastric, and colorectal cancers, which were the three most prevalent cancers in our cohort. As shown in Supplementary Table S7, the PC1 exhibited strong and consistent associations with frailty across all three subtypes. In lung cancer, each SD increase in PC1 corresponded to an adjusted OR of 1.96 (95% CI: 1.59–2.42; *P* < 0.001) for frailty; adjusted ORs were 2.06 (95% CI: 1.60–2.64; *P* < 0.001) for colorectal and 2.10 (95% CI: 1.62–2.72; *P* < 0.001) for gastric cancer. PC2 and PC4 showed weak or nonsignificant associations in most subgroups. Specifically, PC2 lost significance after adjusting for all three types. PC4 showed marginal association in lung cancer (adjusted OR: 1.19, 95% CI: 0.98–1.46; *P* = 0.084), with no significance in colorectal or gastric cancers. The interactions between the PCs were generally non-significant.

As shown in Supplementary Table S8, with pre-frailty as the reference, frailty status was independently associated with OS across all subgroups (lung cancer: adjusted HR = 1.58, 95%CI: 1.24–2.01, *P* < 0.001; colorectal cancer: adjusted HR = 1.86, 95%CI: 1.33–2.61, *P* < 0.001; gastric cancer: adjusted HR = 1.62, 95%CI: 1.19–2.22, *P* = 0.002), with a significant mortality trend across frailty levels (*P* for trend < 0.001). Supplementary Table S9 shows that PC1 remained a significant predictor of OS in all the subgroups, whereas PC2 and PC4 were significant predictors for the selected cancer types.

## Discussion

To the best of our knowledge, this is the first large-scale study involving 2219 older patients with cancer that identified significant associations between frailty and multidimensional biological patterns derived from 18 biomarkers. Through PCA, we identified three clinically meaningful biomarker patterns—malnutrition (PC1), metabolic dysregulation (PC2), and systemic inflammation (PC4)—which not only associated with frailty phenotypes but also independently predicted OS. Additionally, frailty status also significantly contributed to a low survival rate in this population.

Frailty is increasingly prevalent among older patients with cancer, largely due to progressive physiological depletion caused by both the malignancy and its associated treatments. It is estimated that over half of older patients with cancer are either frail or in a pre-frail state.^[20]^ In our cohort, 27.1% of patients were classified as frail, 48.9% as pre-frail, and only 24.0% as non-frail. This distribution aligns with prior evidence that reported a preoperative frailty prevalence of 20.7% among older patients with gastric cancer.^[21]^ Furthermore, our results demonstrate a significant age-related increase in the prevalence of frailty. Frail patients had a higher median age (71.00 years) compared to their pre-frail (69.00 years) and robust (68.00 years) counterparts (*P* < 0.001). The cumulative probability curves indicated a steady increase in the incidence of frailty with advancing age in both sexes.

Routine clinical and laboratory indicators, such as TP, ALB, and BMI, are widely available and cost-effective tools commonly used for risk assessment and prognostication in cancer care. To advance beyond the assessment of isolated biomarkers, we applied PCA to 18 indicators to derive three composite biomarker patterns. Frail patients exhibited significantly higher scores across all three PCs than the pre-frail and non-frail groups, and multivariable logistic regression models further confirmed that each PC was independently associated with frailty. PC1 reflects nutritional depletion and muscle atrophy, which are canonical features of frailty and adverse oncologic outcomes. Sarcopenia, which is a core manifestation of muscle loss, may mediate the relationship between malnutrition and frailty. Indeed, a cross-sectional study of 4122 cancer patients reported that all individuals with sarcopenia also exhibited frailty,^[22]^ whereas a prospective study of hospitalized lung cancer patients in China found a significant co-occurrence of frailty, sarcopenia, and malnutrition among patients aged ≥ 65 years.^[23]^ PC2 captured the metabolic disturbance pattern marked by elevated AST, ALT, bilirubin, LDL, and cholesterol levels. Mechanistically, the features represented by PC2 may partly reflect the hepatic metabolic perturbations that promote muscle loss and frailty through multiple pathways.^[24,25]^ Specifically, tumor burden and treatment-related hepatotoxicity can impair key hepatic functions, leading to hyperammonemia and altered amino acid metabolism, which impair skeletal muscle protein synthesis and promote proteolysis and autophagy, which are well-documented in cirrhosis and experimental models.^[26]^ Hyperammonemia has been shown to induce mitochondrial dysfunction in muscles and activate myostatin-mediated atrophy pathways, and translational studies have demonstrated that reducing ammonia levels can reverse muscle wasting.^[27]^ The significance of AST and ALT levels extends beyond hepatotoxicity. Because they are expressed in extrahepatic tissues, including skeletal and cardiac muscles, and serve as key aminotransferases involved in gluconeogenesis, amino acid turnover, and the malate–aspartate shuttle, these enzymes can reflect systemic catabolic states and metabolic stress. Elsheikh *et al*. showed that in patients with end-stage liver disease, elevated AST and ALT levels indicated not only hepatocellular injury but also systemic metabolic dysfunction, including impaired lipid metabolism and energy production.^[28]^ This process further increases systemic oxidative stress and inflammation, thereby damaging myocyte mitochondrial quality control, accelerating sarcopenia, and ultimately leading to frailty.^[29,30]^ Bilirubin acts as an endogenous antioxidant and is associated with systemic redox balance and metabolic regulation.^[31]^ Elevated bilirubin levels in our cohort further point toward altered redox balance and bile acid homeostasis, which are increasingly recognized as critical factors in the human aging process.^[32]^ PC4, which reflects the inflammatory profile, is characterized by increased levels of white blood cells, neutrophils, and lymphocytes. Recent mechanistic studies have provided strong biological support for a link between systemic inflammation and frailty. Chronic low-grade inflammation, marked by increased circulating cytokines such as IL-6, TNF-α, and IL-1β, promotes muscle catabolism, mitochondrial dysfunction, and impaired myocyte regeneration, thereby accelerating functional decline and the development of frailty.^[29,33]^ Moreover, the activation of the NLRP3 inflammasome has been implicated in age-related inflammation and sarcopenia, and experimental inhibition or genetic deletion of NLRP3 attenuates several aging phenotypes in preclinical models, suggesting a causal role in frailty biology.^[34,35]^ Collectively, these mechanistic findings reinforce our observation that inflammation-related PC is correlated with frailty status in older patients with cancer. Consistently, our previous study demonstrated that an elevated NLR was significantly associated with frailty and that the coexistence of frailty and systemic inflammation conferred the highest mortality risk.^[36]^

In addition, our findings demonstrate that these biological patterns are not only associated with frailty status, but also possess independent prognostic significance, with the exception of PC4. PC4 tended to be associated with worse prognosis, suggesting potential confounding by tumor stage and comorbidities. Furthermore, the interaction terms between PC1 and PC2, PC1 and PC4, and PC2 and PC4 were not statistically significant in either the unadjusted or adjusted models for frailty or in the corresponding Cox regression models assessing survival. These findings suggest that the prognostic effects of the individual components operate independently, implying that the three patterns influence frailty and survival through independent pathways.

In addition to its statistical performance, the PCA model has translational implications. Model comparisons indicated that PC1 achieved a higher C-index than some single markers such as BMI or NLR, underscoring the superior discriminative ability of multidimensional biomarker integration. Because these indicators are routinely measured in oncology practice, PC scores can be easily computed to provide an integrated assessment of the patients' nutritional, metabolic, and inflammatory statuses. Thus, PCA may serve as a bridge between biological data and clinical decision-making, transforming complex biomarker profiles into frailty risk assessment and longitudinal prognostic monitoring for personalized management of older adults with cancer.

Frailty is associated with adverse cancer outcomes, including chemotherapy intolerance, postoperative complications, and increased mortality, across various tumor types and treatment settings.^[37]^ A large prospective cohort study from the UK Biobank demonstrated a stepwise increase in cancer-related mortality as frailty worsened; frail individuals experienced a 39% higher risk of cancer-related death than their non-frail counterparts independent of other risk factors.^[38]^ Comparable findings have been reported among patients undergoing esophageal cancer surgery,^[7]^ gastric cancer surgery,^[8]^ and older patients with breast cancer receiving adjuvant chemotherapy.^[39]^ Consistent with these observations, our study revealed a clear relationship between the severity of frailty and OS in older patients with cancer. A statistically significant trend toward increased mortality risk was observed with advancing frailty status. Moreover, frailty was stratified into non-frail, pre-frail, and frail categories, showing that frail individuals had a 68% increased risk of mortality compared with pre-frail individuals. This compelling evidence underscores the critical importance of systematically monitoring the frailty status in older patients with cancer to prevent further deterioration.

Subgroup analyses revealed that although the associations among PCs, frailty, and OS were directionally consistent across major tumor types, their magnitudes differed. The malnutrition-related component (PC1) showed strong and consistent associations with both frailty and survival in lung, gastric, and colorectal cancers, highlighting malnutrition as a shared biological basis linking frailty to poor prognosis, regardless of cancer type. In contrast, the metabolic (PC2) and inflammatory (PC4) components exhibited more variable relationships. Both PCs showed weak or non-significant associations with frailty in most subgroups, suggesting that metabolic and inflammatory pathways may influence frailty in a tumor-specific manner. Notably, PC2 and PC4 were significantly associated with survival in lung cancer, but not in gastric or colorectal cancers, implying that these processes may play a particularly important role in pulmonary malignancies. Importantly, frailty remained an independent predictor of OS across all the subgroups, reinforcing its robust prognostic relevance.

This study has several limitations. First, the cross-sectional design of the frailty assessment at a single time point precludes the evaluation of longitudinal relationships between biomarker patterns and frailty progression. Therefore, the present findings should be interpreted as associative, rather than causal. Future work incorporating longitudinal biomarker measurements is warranted to capture the dynamic changes in both biomarkers and frailty status over time. Such an approach may help disentangle causal pathways, identify early biological signals of impending frailty, and improve individualized risk stratification. Additionally, sensitivity analyses will be performed to explore whether the duration of cancer diagnosis influences the associations between the principal components and frailty. Second, despite the multicenter design and large sample size, the cohort was drawn exclusively from Chinese hospitals, which may have limited the external validity of our findings across diverse populations. For instance, differences in the prevalence of sarcopenia between Chinese and Western populations highlight the need for future studies involving multiple ethnic cohorts. Third, the lack of detailed cytokine profiles and other metabolic markers in our data limits our mechanistic understanding of frailty pathophysiology. Future studies incorporating comprehensive biomarker analyses are warranted to elucidate these mechanisms. Fourth, although we adopted the conceptual framework of Fried's frailty phenotype, three of the five components were assessed using data from the QLQ-C30. Future studies incorporating objective physical performance measures such as gait speed are warranted to validate these findings. Finally, although extensive adjustments were made for clinical confounders, the possibility of residual confounding due to unmeasured variables cannot be entirely excluded.

## Conclusion

In this large multicenter cohort of older adults with cancer, biological patterns reflecting nutritional deficits, metabolic dysregulation, and systemic inflammation were significantly associated with frailty. Furthermore, these patterns independently predicted overall survival. In addition, frailty demonstrated a strong independent association with decreased survival in older patients with cancer.

## Supplementary Information

Supplementary materials are only available at the official site of the journal (www.intern-med.com).

## Supplementary Material

Supplementary Material Details
