# Supplementary material for: Multidimensional physiological impairments are associated with frailty and survival in older patients with cancer
Source: J Transl Int Med. 2026 Aug 24;14(4):589–98. doi: 10.1515/jtim-2026-0057 (PMC13509152; doi:10.1515/jtim-2026-0057)
Supplement: Supplementary file 1 [file jtim-2026-0057_sm.pdf]

Supplementary materials

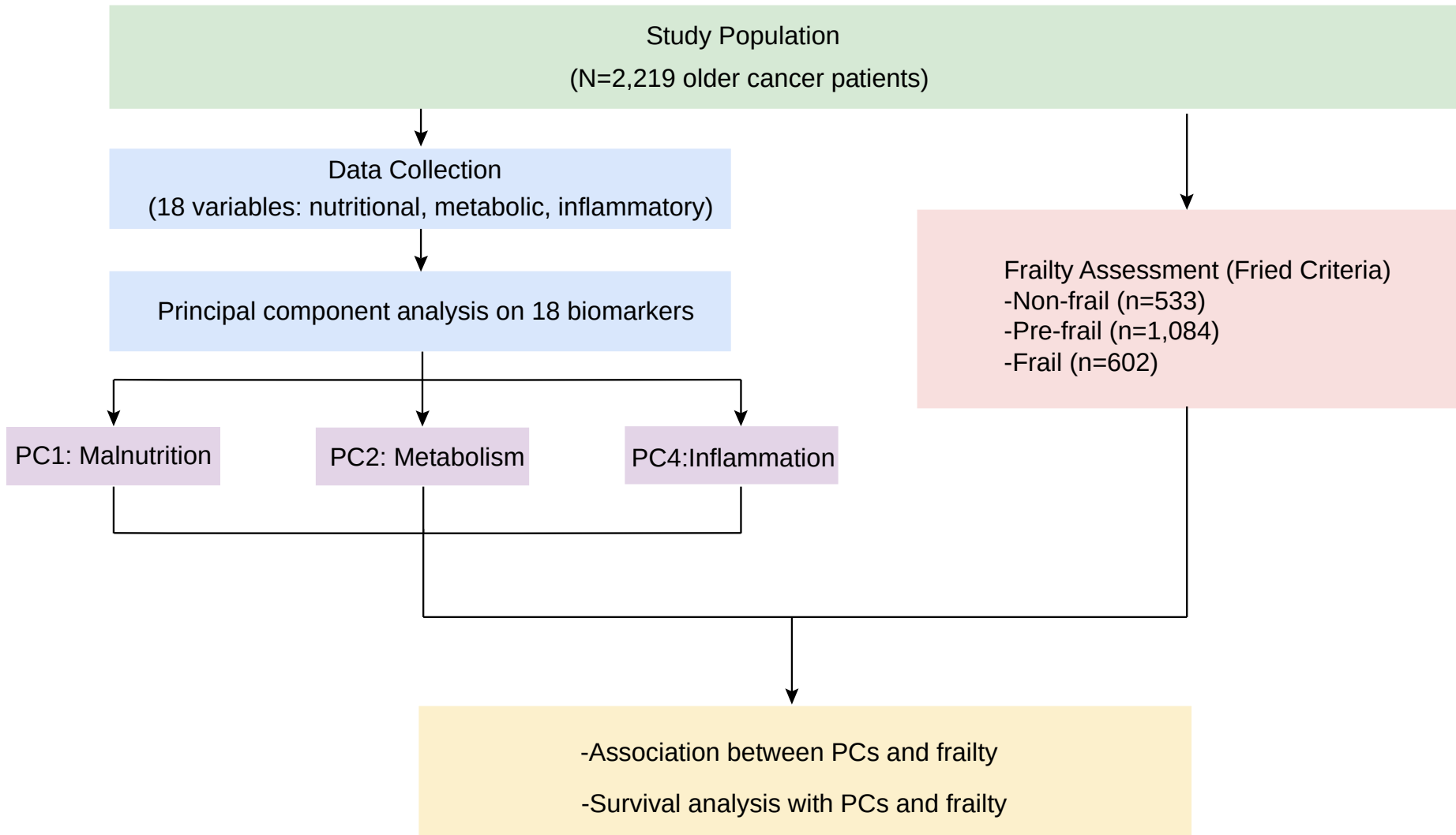

Supplementary Figure S1: Flow chart.

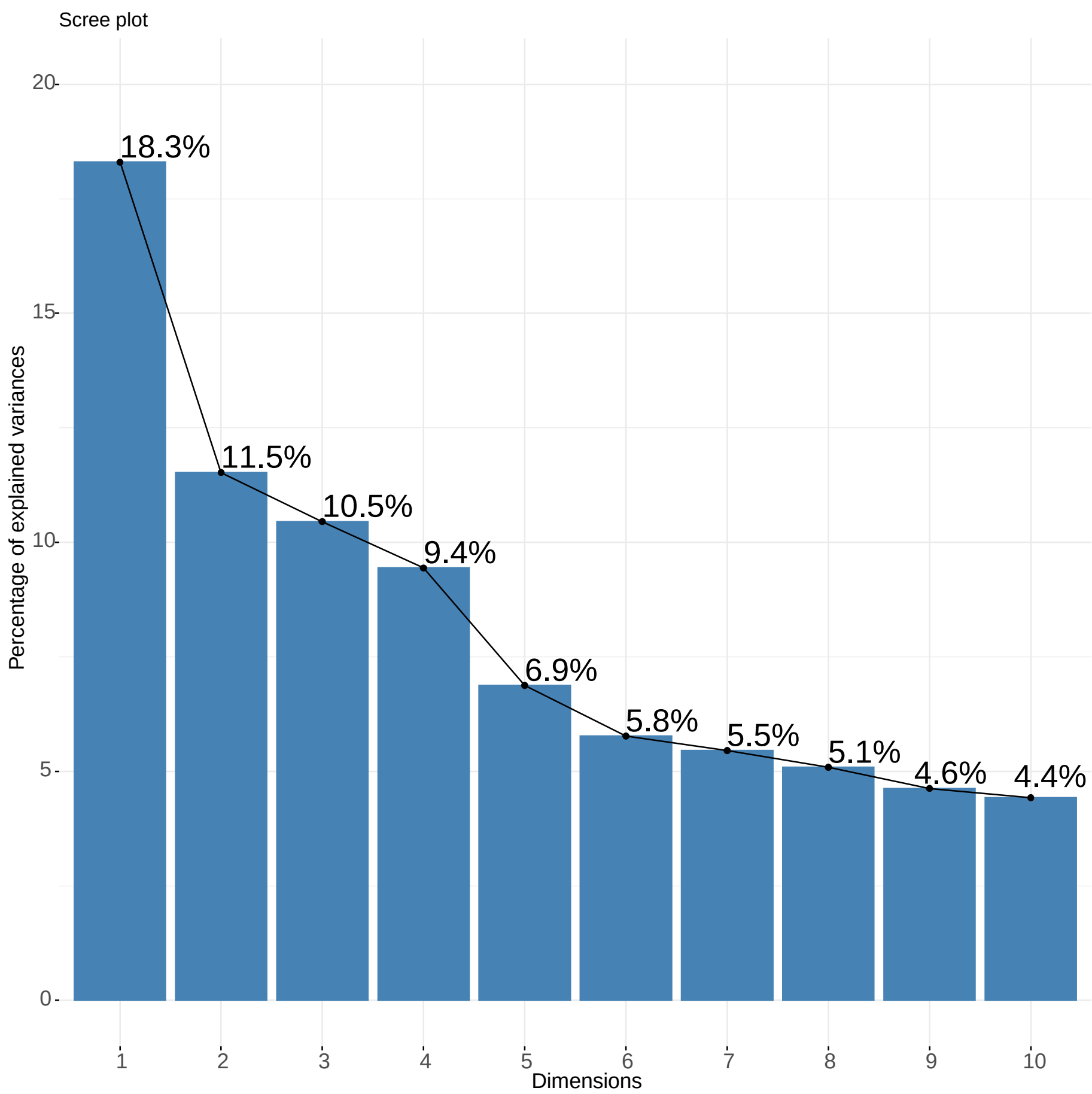

Supplementary Figure S2: Scree plot of eigenvalues from principal component analysis (PCA) of the top 10 principal components.

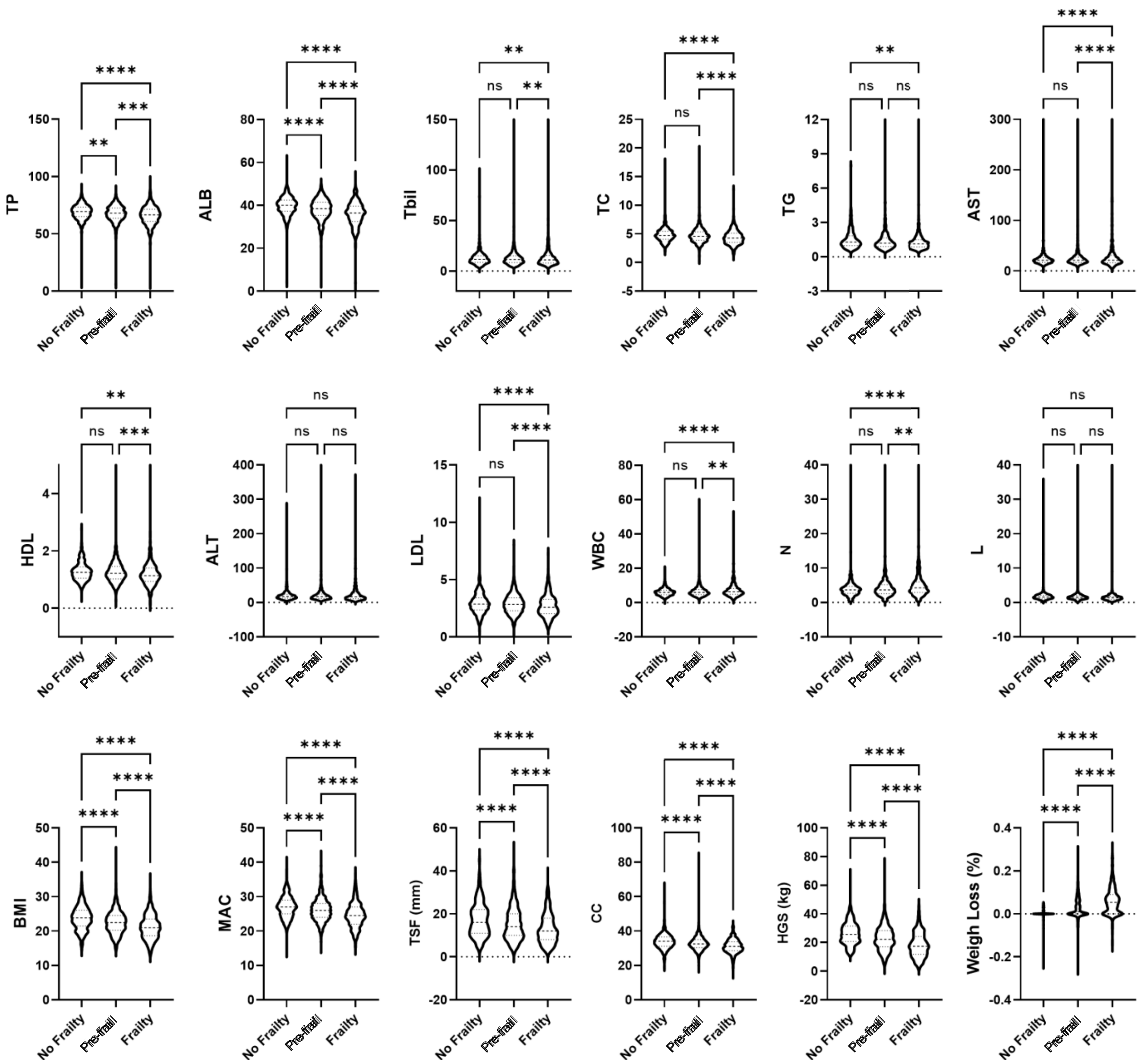

Supplementary Figure S3: 18 variables encompassing nutritional, metabolic, and inflammatory biomarkers by frailty status among 2219 patients. Data are shown as boxplots; differences between frailty groups were assessed using Student's *t*-test. ns, not significant; $^{*}P < 0.05$; $^{**}P < 0.01$; $^{***}P < 0.001$; $^{****}P < 0.0001$.

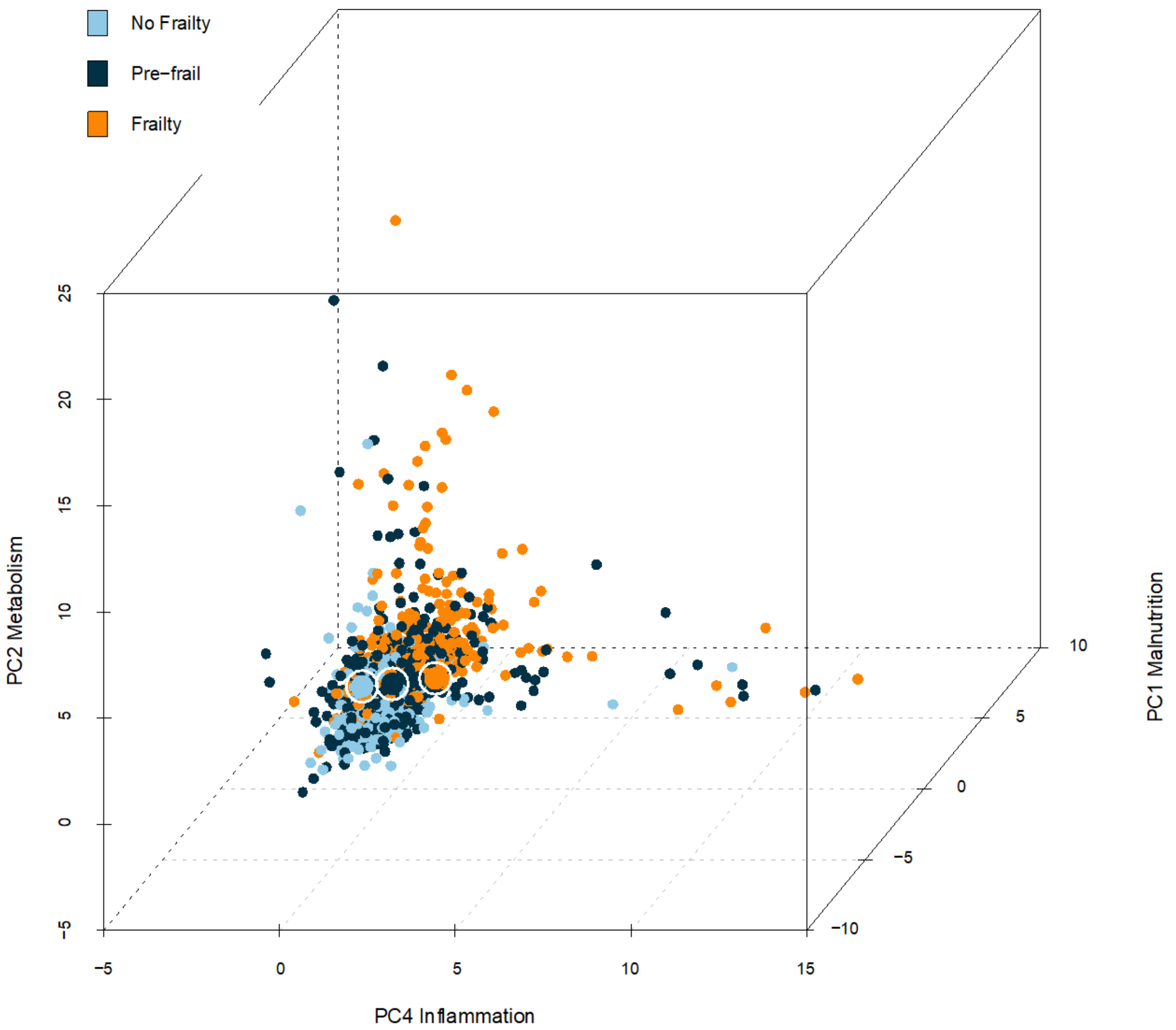

Supplementary Figure S4: Three-dimensional principal component plot illustrating the distribution of frailty phenotypes based on nutritional, metabolic, and inflammatory biomarkers. This 3D scatter plot visualizes the distribution of 2,219 elderly cancer patients according to the three principal components (PC1, PC2, PC4), derived from 18 variables. Each point represents an individual, color-coded by frailty status: robust, pre-frail, and frail. PC1 primarily reflects malnutrition-related features, PC2 corresponds to metabolic alterations, and PC4 captures inflammatory markers. The spatial separation among groups suggests distinct biological profiles underlying frailty phenotypes. PC, principal component.

TABLES

Supplementary Table S1: Operational definitions of Fried's frailty phenotype and corresponding adaptations used in this study.

Frailty component	Original Fried phenotype	Operationalization in current study
Shrinking	Unintentional weight loss ⩾ 10 pounds or ⩾ 5% of body weight in the past year	Unintentional weight loss ⩾ 5% of body weight, consistent with Fried criterion
Weakness	Handgrip strength (HGS) in lowest 20%, adjusted for sex and BMI	HGS measured with calibrated dynamometer; lowest sex-adjusted 20% classified as "weak", consistent with Fried criterion
Slowness	Slowest 20% of 4-m walking time, adjusted for sex and standing height	Approximated by EORTC QLQ-C30 item 3: "Do you have any trouble taking a short walk outside of the house?" (Response score ⩾ 2 = "present")
Exhaustion	Two CES-D items ("I felt that everything I did was an effort" or "I could not get going") ⩾ 3–4 days /week	Mapped to EORTC QLQ-C30 item 12: "Have you felt weak?" (Response score ⩾ 2 = "present")
Low physical activity	Weekly energy expenditure (kcal) in lowest 20% by sex (<383 kcal/week for men or <270 kcal/week for women)	Approximated by EORTC QLQ-C30 item 6: "Are you limited in doing either your work or household jobs?" (Response score ⩾ 2 = "present")

Supplementary Table S2: Variance Inflation Factors (VIFs) for predictors in regression models.

Logistic Regression

Variable	PC1 model			PC2 model			PC4 model		
	VIF	Tolerance (1/VIF)	Interpretation	VIF	Tolerance (1/VIF)	Interpretation	VIF	Tolerance (1/VIF)	Interpretation
Age, years	1.08	0.926	No collinearity	1.07	0.933	No collinearity	1.08	0.929	No collinearity
Gender									
Male	Reference								
Female	1.45	0.690	No collinearity	1.45	0.689	No collinearity	1.46	0.685	No collinearity
Chronic Disease									
No	Reference								
Yes	1.07	0.938	No collinearity	1.05	0.953	No collinearity	1.05	0.953	No collinearity
Smoking									
No	Reference								
Yes	1.54	0.648	No collinearity	1.53	0.652	No collinearity	1.53	0.652	No collinearity
Drinking									
No	Reference								
Yes	1.23	0.813	No collinearity	1.23	0.813	No collinearity	1.23	0.815	No collinearity
Type									
Lung cancer	Reference								
Gastric cancer	1.60	0.625	No collinearity	1.53	0.656	No collinearity	1.52	0.656	No collinearity
Hepatobiliary and pancreatic cancers	1.24	0.809	No collinearity	1.22	0.821	No collinearity	1.20	0.835	No collinearity
Esophagus cancer	1.27	0.790	No collinearity	1.25	0.799	No collinearity	1.25	0.798	No collinearity
Colorectal cancer	1.63	0.614	No collinearity	1.61	0.621	No collinearity	1.61	0.622	No collinearity
Other cancers	1.36	0.736	No collinearity	1.36	0.734	No collinearity	1.36	0.734	No collinearity
Tumor stage									
Ⅰ / Ⅱ	Reference								
Ⅲ/Ⅳ	1.49	0.671	No collinearity	1.50	0.667	No collinearity	1.50	0.667	No collinearity
Metastasis									
No	Reference								
Yes	1.56	0.642	No collinearity	1.57	0.638	No collinearity	1.56	0.64	No collinearity
Surgery									
No	Reference								
Yes	1.46	0.686	No collinearity	1.44	0.697	No collinearity	1.43	0.70	No collinearity
Chemotherapy									
No	Reference								
Yes	1.11	0.903	No collinearity	1.12	0.896	No collinearity	1.12	0.896	No collinearity
Radiotherapy									
No	Reference								
Yes	1.09	0.917	No collinearity	1.10	0.907	No collinearity	1.11	0.904	No collinearity
PC1	1.10	0.909	No collinearity	/	/	/	/	/	/
PC2	/	/	/	1.05	0.957	No collinearity	/	/	/
PC4	/	/	/	/	/	/	1.03	0.969	No collinearity

Cox Regression

Variable	PC1 model			PC2 model			PC4 model		
	VIF	Tolerance (1/VIF)	Interpretation	VIF	Tolerance (1/VIF)	Interpretation	VIF	Tolerance (1/VIF)	Interpretation
Age, years	1.12	0.896	No collinearity	1.09	0.922	No collinearity	1.10	0.913	No collinearity
Gender									
Male	Reference								
Female	1.39	0.721	No collinearity	1.40	0.712	No collinearity	1.41	0.711	No collinearity
Chronic Disease									
No	Reference								
Yes	1.08	0.93	No collinearity	1.06	0.943	No collinearity	1.06	0.941	No collinearity
Smoking									
No	Reference								
Yes	1.48	0.676	No collinearity	1.49	0.671	No collinearity	1.49	0.673	No collinearity
Drinking									
No	Reference								
Yes	1.25	0.801	No collinearity	1.25	0.799	No collinearity	1.25	0.802	No collinearity
Type									
Lung cancer	Reference								
Gastric cancer	1.54	0.648	No collinearity	1.48	0.678	No collinearity	1.47	0.681	No collinearity
Hepatobiliary and pancreatic cancers	1.24	0.805	No collinearity	1.28	0.784	No collinearity	1.22	0.818	No collinearity
Esophagus cancer	1.25	0.802	No collinearity	1.23	0.813	No collinearity	1.23	0.813	No collinearity
Colorectal cancer	1.50	0.666	No collinearity	1.51	0.663	No collinearity	1.51	0.663	No collinearity
Other cancers	1.25	0.800	No collinearity	1.25	0.800	No collinearity	1.25	0.799	No collinearity
Tumor stage									
Ⅰ / Ⅱ	Reference								
Ⅲ/Ⅳ	1.36	0.736	No collinearity	1.36	0.734	No collinearity	1.36	0.733	No collinearity
Metastasis									
No	Reference								
Yes	1.47	0.681	No collinearity	1.47	0.680	No collinearity	1.47	0.68	No collinearity
Surgery									
No	Reference								
Yes	1.44	0.693	No collinearity	1.45	0.692	No collinearity	1.44	0.695	No collinearity
Chemotherapy									
No	Reference								
Yes	1.12	0.89	No collinearity	1.12	0.889	No collinearity	1.13	0.886	No collinearity
Radiotherapy									
No	Reference								
Yes	1.13	0.883	No collinearity	1.14	0.875	No collinearity	1.15	0.872	No collinearity
PC1	1.12	0.894	No collinearity	/	/	/	/	/	/
PC2	/	/	/	1.07	0.930	No collinearity	/	/	/
PC4	/	/	/	/	/	/	1.04	0.966	No collinearity

Supplementary Table S3: Baseline characteristics.

Variables	Overall (*n* = 2219)	No Frailty (*n* = 533)	Prefrail (*n* = 1084)	Frailty (*n* = 602)	*P* value
Gender (%)					
Male	1416 (63.8)	331 (62.1)	697 (64.3)	388 (64.5)	0.640
Female	803 (36.2)	202 (37.9)	387 (35.7)	214 (35.5)	
Age, years, (median [IQR])	69.00 [66.00, 74.00]	68.00 [66.00, 72.00]	69.00 [66.00, 73.00]	71.00 [67.00, 76.00]	<0.001
BMI (%)					
< 18.5	295 (13.3)	31 (5.8)	122 (11.3)	142 (23.6)	<0.001
18.5-24	1191 (53.7)	244 (45.8)	619 (57.2)	328 (54.5)	
24-28	596 (26.9)	197 (37.0)	284 (26.2)	115 (19.1)	
⩾ 28	136 (6.1)	61 (11.4)	58 (5.4)	17 (2.8)	
Chronic Disease (%)					
No	1113 (50.2)	283 (53.1)	557 (51.4)	273 (45.3)	0.018
Yes	1106 (49.8)	250 (46.9)	527 (48.6)	329 (54.7)	
Smoking (%)					
No	1147 (51.7)	283 (53.1)	548 (50.6)	316 (52.5)	0.566
Yes	1072 (48.3)	250 (46.9)	536 (49.4)	286 (47.5)	
Drinking (%)					
No	1802 (81.2)	424 (79.5)	872 (80.4)	506 (84.1)	0.102
Yes	417 (18.8)	109 (20.5)	212 (19.6)	96 (15.9)	
Tea (%)					
No	1640 (73.9)	392 (73.5)	795 (73.3)	453 (75.2)	0.677
Yes	579 (26.1)	141 (26.5)	289 (26.7)	149 (24.8)	
Tumor Type (%)					
Lung cancer	698 (31.5)	181 (34.0)	328 (30.3)	189 (31.4)	<0.001
Gastric cancer	373 (16.8)	54 (10.1)	197 (18.2)	122 (20.3)	
Hepatobiliary and pancreatic cancers	133 (6.0)	26 (4.9)	58 (5.4)	49 (8.1)	
Esophagus cancer	185 (8.3)	28 (5.3)	102 (9.4)	55 (9.1)	
Colorectal cancer	484 (21.8)	108 (20.3)	255 (23.5)	121 (20.1)	
Other cancers	346 (15.6)	136 (25.5)	144 (13.3)	66 (11.0)	
TNM Stage (%)					
I	212 (9.6)	71 (13.3)	103 (9.5)	38 (6.3)	<0.001
II	447 (20.1)	127 (23.8)	228 (21.0)	92 (15.3)	
III	586 (26.4)	162 (30.4)	294 (27.1)	130 (21.6)	
IV	974 (43.9)	173 (32.5)	459 (42.3)	342 (56.8)	
Metastasis (%)					
No	1308 (58.9)	375 (70.4)	653 (60.2)	280 (46.5)	<0.001
Yes	911 (41.1)	158 (29.6)	431 (39.8)	322 (53.5)	
Chemotherapy					
No	789 (35.6)	194 (36.4)	363 (33.5)	232 (38.5)	0.104
Yes	1430 (64.4)	339 (63.6)	721 (66.5)	370 (61.5)	
Radiotherapy					
No	1914 (86.3)	453 (85.0)	937 (86.4)	524 (87.0)	0.587
Yes	305 (13.7)	80 (15.0)	147 (13.6)	78 (13.0)	
Surgery					
No	780 (35.2)	177 (33.2)	363 (33.5)	240 (39.9)	0.018
Yes	1439 (64.8)	356 (66.8)	721 (66.5)	362 (60.1)	
NLR (%)					
<3	1366 (61.6)	385 (72.2)	687 (63.4)	294 (48.8)	<0.001
⩾3	853 (38.4)	148 (27.8)	397 (36.6)	308 (51.2)	
Functional assessment, (median [IQR])	86.00 [75.50, 95.33]	97.33 [92.00, 100.00]	86.67 [79.33, 94.00]	69.00 [56.42, 78.58]	<0.001
Global Health, (median [IQR])	66.67 [50.00, 83.33]	83.33 [66.67, 91.67]	66.67 [50.00, 75.00]	50.00 [33.33, 66.67]	<0.001
Symptom assessment, (median [IQR])	10.49 [3.70, 18.52]	3.70 [0.00, 7.41]	9.88 [3.70, 16.05]	21.30 [14.81, 29.63]	<0.001
EORTC QLQ-C30, (median [IQR])	53.12 [47.59, 59.23]	60.05 [53.95, 64.63]	53.44 [49.26, 58.75]	47.26 [40.84, 52.46]	<0.001

BMI: Body Mass Index; TNM: Tumor/Node/Metastasis; NLR: Neutrophil-to-Lymphocyte Ratio; EORTC QLQ-C30: European Organization for Research and Treatment of Cancer Quality of Life Questionnaire - Core 30.

Supplementary Table S4: Prognostic impact of nutritional, metabolic, and inflammatory components on overall survival (OS).

Survival-per SD	HR (95%CI)	*P* value	Adjusted HR (95%CI)	*P* value
PC1.malnutrition	1.43 (1.35, 1.51)	<0.001	1.30 (1.22, 1.38)	<0.001
PC2.metabolism	1.18 (1.12, 1.24)	<0.001	1.11 (1.05, 1.17)	<0.001
PC4.inflammation	1.08 (1.02, 1.14)	0.014	1.06 (1.00, 1.12)	0.061
PC1 × PC2 interaction	0.98 (0.95, 1.02)	0.320	1.01 (0.97, 1.05)	0.715
PC1 × PC4 interaction	1.04 (0.99, 1.09)	0.143	1.02 (0.96, 1.08)	0.523
PC2 × PC4 interaction	1.02 (0.98, 1.07)	0.323	1.01 (0.97, 1.06)	0.601

Adjusted for age, sex, chronic disease, smoking, drinking, tumor type, tumor stage, metastasis, surgery, chemotherapy, radiotherapy. PC, principal component.

Supplementary Table S5: Cox regression analyses of baseline characteristics for OS in older patients with cancer.

Variables	Univariate		Multivariate	
	HR (95%CI)	*P* value	HR (95%CI)	*P* value
Gender				
Male	Reference			
Female	0.67 (0.58, 0.77)	<0.001	0.77 (0.65, 0.90)	0.001
Age, years	1.03 (1.02, 1.04)	<0.001	1.02 (1.01, 1.03)	0.005
BMI, kg/m^2	0.92 (0.90, 0.93)	<0.001	0.96 (0.94, 0.97)	0.001
Chronic Disease				
No	Reference			
Yes	0.95 (0.84, 1.08)	0.450		
Smoking				
No	Reference			
Yes	1.28 (1.13, 1.45)	<0.001	0.93 (0.80, 1.08)	0.320
Drinking				
No	Reference			
Yes	1.10 (0.94, 1.29)	0.228		
Tea				
No	Reference			
Yes	1.06 (0.92, 1.22)	0.422		
Tumor Type				
Lung cancer	Reference			
Gastric cancer	1.00 (0.84, 1.20)	0.962	1.22 (1.00, 1.48)	0.045
Hepatobiliary and pancreatic cancers	1.50 (1.17, 1.92)	0.001	1.79 (1.39, 2.31)	0.001
Esophagus cancer	1.21 (0.97, 1.52)	0.094	1.48 (1.17, 1.86)	0.001
Colorectal cancer	0.67 (0.56, 0.80)	<0.001	0.83 (0.68, 1.01)	0.062
Other cancers	0.39 (0.31, 0.49)	<0.001	0.54 (0.43, 0.69)	0.001
TNM Stage				
Ⅰ / Ⅱ	Reference			
Ⅲ/Ⅳ	3.18 (2.68, 3.77)	<0.001	2.02 (1.66, 2.47)	0.001

Metastasis				
No	Reference			
Yes	2.88 (2.53, 3.27)	<0.001	1.60 (1.36, 1.87)	0.001
Chemotherapy				
No	Reference			
Yes	1.32 (1.15, 1.51)	<0.001	1.25 (1.08, 1.44)	0.003
Radiotherapy				
No	Reference			
Yes	1.06 (0.89, 1.27)	0.489		
Surgery				
No	Reference			
Yes	0.46 (0.41, 0.53)	<0.001	0.72 (0.61, 0.83)	0.001
NLR (%)				
<3	Reference			
⩾3	1.70 (1.50, 1.93)	<0.001	1.31 (1.15, 1.49)	0.001
EORTC QLQ-C30	0.95 (0.95, 0.96)	<0.001	0.97 (0.96, 0.97)	0.001

BMI: Body Mass Index; TNM: Tumor/Node/Metastasis; NLR: Neutrophil-to-Lymphocyte Ratio; EORTC QLQ-C30: European Organization for Research and Treatment of Cancer Quality of Life Questionnaire - Core 30.

Supplementary Table S6: C-index of variables for survival prediction in older patients with cancer, including gender, age, BMI, tumor type, TNM stage, metastasis, chemotherapy, surgery, NLR, albumin, EORTC QLQ-C30, PC1.malnutrition, PC2.metabolism, and frailty.

**Variables**	**C-index**	**95% Confidence interval**
Gender	0.541	0.525, 0.557
Age	0.542	0.523, 0.562
BMI	0.591	0.572-0.609
Tumor type	0.571	0.553, 0.589
TNM stage	0.609	0.596, 0.622
Metastasis	0.629	0.613, 0.645
Chemotherapy	0.528	0.512, 0.544
Surgery	0.589	0.573, 0.605
NLR	0.577	0.561, 0.594
EORTC QLQ-C30	0.618	0.599, 0.637
PC1.malnutrition	0.620	0.602, 0.639
PC2.metabolism	0.524	0.503, 0.544
Frailty	0.641	0.623, 0.659

C-index: Concordance index.

Supplementary Table S7: Associations between principal components and frailty status in cancer subgroups (lung, colorectal, and gastric cancer).

Lung cancer				
**Frailty-per SD**	**OR (95%CI)**	***P* value**	**Adjusted OR (95%CI)**	***P* value**
PC1.malnutrition	2.03 (1.67, 2.47)	<0.001	1.96 (1.59, 2.42)	<0.001
PC2.metabolism	1.24 (1.03, 1.49)	0.024	1.17 (0.97, 1.43)	0.106
PC4.inflammation	1.13 (0.94, 1.37)	0.202	1.19 (0.98, 1.46)	0.084
PC1 × PC2 interaction	1.04 (0.83, 1.30)	0.725	1.10 (0.88, 1.38)	0.406
PC1 × PC4 interaction	0.88 (0.70, 1.12)	0.305	0.86 (0.67, 1.11)	0.240
PC2 × PC4 interaction	1.20 (0.96, 1.50)	0.106	1.16 (0.93, 1.45)	0.184
**Colorectal Cancer**				
**Frailty-per SD**	**OR (95%CI)**	***P* value**	**Adjusted OR (95%CI)**	***P* value**
PC1.malnutrition	2.19 (1.73, 2.78)	<0.001	2.06 (1.60, 2.64)	<0.001
PC2.metabolism	1.10 (0.92, 1.31)	0.321	1.08 (0.89, 1.31)	0.423
PC4.inflammation	0.96 (0.75, 1.22)	0.715	0.94 (0.72, 1.23)	0.667
PC1 × PC2 interaction	1.08 (0.84, 1.39)	0.552	1.05 (0.85, 1.30)	0.659
PC1 × PC4 interaction	1.18 (0.89, 1.56)	0.257	1.16 (0.86, 1.56)	0.339
PC2 × PC4 interaction	1.13 (0.97, 1.32)	0.104	1.12 (0.96, 1.30)	0.157
**Gastric cancer**				
**Frailty-per SD**	**OR (95%CI)**	***P* value**	**Adjusted OR (95%CI)**	***P* value**
PC1.malnutrition	2.03 (1.59, 2.58)	<0.001	2.10 (1.62, 2.72)	<0.001
PC2.metabolism	1.14 (0.94, 1.38)	0.173	1.12 (0.92, 1.37)	0.265
PC4.inflammation	1.03 (0.89, 1.20)	0.662	1.05 (0.90, 1.23)	0.537
PC1 × PC2 interaction	0.94 (0.81, 1.08)	0.356	0.93 (0.80, 1.08)	0.372
PC1 × PC4 interaction	1.16 (0.86, 1.55)	0.329	1.20 (0.87, 1.64)	0.272
PC2 × PC4 interaction	1.07 (0.93, 1.24)	0.334	1.03 (0.89, 1.20)	0.689

Adjusted for age, sex, chronic disease, smoking, drinking, tumor stage, metastasis, surgery, chemotherapy, radiotherapy.

Supplementary Table S8: Association of frailty status with overall survival (OS) in cancer subgroups (lung, colorectal, and gastric cancer).

Variables	HR (95%CI)	*P* value	Adjusted HR (95%CI)	*P* value
**Lung cancer**				
No Frailty	0.73 (0.55, 0.97)	0.031	0.72 (0.53, 0.96)	0.025
Pre-frail		reference		reference
Frailty	2.04 (1.62, 2.58)	<0.001	1.58 (1.24, 2.01)	<0.001
P for trend		<0.001		<0.001
**Colorectal Cancer**				
**Variables**	**HR (95%CI)**	***P* value**	**Adjusted HR (95%CI)**	***P* value**
No Frailty	0.69 (0.45, 1.05)	0.086	0.89 (0.58, 1.38)	0.603
Pre-frail		reference		reference
Frailty	2.00 (1.44, 2.76)	<0.001	1.86 (1.33, 2.61)	<0.001
P for trend		<0.001		<0.001
**Gastric cancer**				
**Variables**	**HR (95%CI)**	***P* value**	**Adjusted HR (95%CI)**	***P* value**
No Frailty	0.70 (0.44, 1.12)	0.136	0.88 (0.54, 1.42)	0.595
Pre-frail		reference		reference
Frailty	1.82 (1.35, 2.47)	<0.001	1.62 (1.19, 2.22)	0.002
P for trend		<0.001		0.002

Adjusted for age, sex, chronic disease, smoking, drinking, tumor stage, metastasis, surgery, chemotherapy, radiotherapy.

Supplementary Table S9: Prognostic impact of nutritional, metabolic, and inflammatory components on overall survival (OS) in cancer subgroups (lung, colorectal, and gastric cancer).

Lung cancer				
**Survival-per SD**	**HR (95%CI)**	***P* value**	**Adjusted HR (95%CI)**	***P* value**
PC1.malnutrition	1.41 (1.26, 1.57)	<0.001	1.27 (1.13, 1.42)	<0.001
PC2.metabolism	1.12 (1.01, 1.25)	0.035	1.12 (1.01, 1.25)	0.033
PC4.inflammation	1.13 (1.00, 1.27)	0.045	1.15 (1.02, 1.30)	0.019
PC1 × PC2 interaction	0.91 (0.83, 1.00)	0.043	0.94 (0.85, 1.03)	0.197
PC1 × PC4 interaction	0.98 (0.86, 1.10)	0.680	0.93 (0.82, 1.05)	0.264
PC2 × PC4 interaction	1.05 (0.92, 1.19)	0.465	1.07 (0.95, 1.21)	0.254
**Colorectal Cancer**				
**Survival-per SD**	**HR (95%CI)**	***P* value**	**Adjusted HR (95%CI)**	***P* value**
PC1.malnutrition	1.32 (1.14, 1.52)	<0.001	1.26 (1.08, 1.47)	0.003
PC2.metabolism	1.26 (1.12, 1.42)	<0.001	1.14 (1.00, 1.29)	0.049
PC4.inflammation	1.11 (0.93, 1.32)	0.241	1.02 (0.86, 1.20)	0.848
PC1 × PC2 interaction	0.97 (0.90, 1.04)	0.334	1.00 (0.92, 1.09)	0.992
PC1 × PC4 interaction	1.15 (0.98, 1.36)	0.095	1.01 (0.86, 1.18)	0.935
PC2 × PC4 interaction	0.98 (0.91, 1.06)	0.680	0.96 (0.89, 1.04)	0.324
**Gastric cancer**				
**Survival-per SD**	**HR (95%CI)**	***P* value**	**Adjusted HR (95%CI)**	***P* value**
PC1.malnutrition	1.26 (1.10, 1.44)	<0.001	1.23 (1.06, 1.42)	0.006
PC2.metabolism	1.14 (1.02, 1.27)	0.024	1.12 (0.99, 1.27)	0.078
PC4.inflammation	1.09 (0.99, 1.19)	0.070	1.09 (0.99, 1.21)	0.074
PC1 × PC2 interaction	1.05 (0.97, 1.13)	0.256	1.04 (0.95, 1.14)	0.407
PC1 × PC4 interaction	1.04 (0.96, 1.13)	0.296	1.06 (0.97, 1.17)	0.207
PC2 × PC4 interaction	1.04 (0.95, 1.13)	0.400	0.98 (0.90, 1.08)	0.703

Adjusted for age, sex, chronic disease, smoking, drinking, tumor stage, metastasis, surgery, chemotherapy, radiotherapy.
